# Gene Expression Music Algorithm-Based Characterization of the Ewing Sarcoma Stem Cell Signature

**DOI:** 10.1155/2016/7674824

**Published:** 2016-06-30

**Authors:** Martin Sebastian Staege

**Affiliations:** University Clinic and Polyclinic for Child and Adolescent Medicine, Martin Luther University of Halle-Wittenberg, 06120 Halle (Saale), Germany

## Abstract

Gene Expression Music Algorithm (GEMusicA) is a method for the transformation of DNA microarray data into melodies that can be used for the characterization of differentially expressed genes. Using this method we compared gene expression profiles from endothelial cells (EC), hematopoietic stem cells, neuronal stem cells, embryonic stem cells (ESC), and mesenchymal stem cells (MSC) and defined a set of genes that can discriminate between the different stem cell types. We analyzed the behavior of public microarray data sets from Ewing sarcoma (“Ewing family tumors,” EFT) cell lines and biopsies in GEMusicA after prefiltering DNA microarray data for the probe sets from the stem cell signature. Our results demonstrate that individual Ewing sarcoma cell lines have a high similarity to ESC or EC. Ewing sarcoma cell lines with inhibited Ewing sarcoma breakpoint region 1-Friend leukemia virus integration 1 (EWSR1-FLI1) oncogene retained the similarity to ESC and EC. However, correlation coefficients between GEMusicA-processed expression data between EFT and ESC decreased whereas correlation coefficients between EFT and EC as well as between EFT and MSC increased after knockdown of EWSR1-FLI1. Our data support the concept of EFT being derived from cells with features of embryonic and endothelial cells.

## 1. Introduction

The stem cell phenotype of cancer cells can be the consequence of the malignant transformation that led to* de novo* acquisition of a stem cell-like phenotype or this phenotype can be reminiscent of a normal stem cell that serves as the cell of origin for the cancer cells. In both cases the gene expression profile of the cancer cells will show similarities to the gene expression profile of stem cells. Characterization of this stem cell signature can be useful for the identification of new target structures and might also give hints about the histogenetic origin of cancer cells in cases where the cell of origin has not been identified.

Ewing sarcoma (or the “Ewing family of tumors,” EFT) is an interesting model for a tumor entity with uncertain cell of origin that might be derived from stem cells. Gene expression data suggest a relationship between EFT and endothelial cells, neuroectodermal cells, or mesenchymal stem cells [1–4]. The majority of EFT carry chromosomal translocations leading to gene fusions between members of the TET (translocated in liposarcoma, Ewing sarcoma breakpoint region 1, TATA box binding protein-associated factor) family of RNA binding proteins and the ETS (avian erythroblastosis virus E26 oncogene homolog) family of transcription factors (reviewed in [5]). In most cases, the TET family member EWSR1 (Ewing sarcoma breakpoint region 1) is fused to the ETS family member FLI1 (Friend leukemia virus integration 1). Ewing proposed that EFT are of endothelial origin [6]. Afterwards, a neuroectodermal origin was suggested by the observation of neuronal marker expression in EFT. Indeed, expression of the EFT specific EWSR1-FLI1 oncogene in neuroblastoma cells can induce an EFT-like phenotype [7]. However, expression of the oncogene in nonneural cells can induce expression of neuronal markers, suggesting that the neuronal phenotype might be partially a consequence of oncogene expression [8]. In addition to neuroectodermal cells, mesenchymal stem cells (MSC) have been discussed as cells of origin for EFT [1, 2, 9–11]. However, the gene expression profile of EWSR1-FLI1 transgenic MSC is not completely identical to the gene expression profile of EFT. MSC are a heterogeneous population of stem cells and the activity of TET-ETS oncofusion proteins is influenced by the host cell type [12, 13]. Therefore, it seems possible that the final phenotype of EFT cells is influenced not only by the TET-ETS fusion type but also by the affected stem cell subpopulation.

Recently, we demonstrated that the transformation of gene expression data into melodies can be used for the “musical” analysis of these data and that the Gene Expression Music Algorithm (GEMusicA) allows the discrimination between samples with different biological behavior [14]. For instance, GEMusicA can be used for the discrimination between different tumor entities or for the discrimination between tumor cells and their normal counterparts [14]. GEMusicA is an alternative method to more conventional methods of microarray data analysis. The outputs of GEMusiA analyses are sound files as well as the corresponding musical scores which can be used for visual presentation of the data. Alternatively, the sound files can be used directly for acoustical data presentation. GEMuiscA preferentially enriches probe sets with high signal intensities which are more likely to have a high impact on the phenotype of a cell [14]. The generated melodies are highly specific for the individual samples and high-pitched notes directly indicate genes with high expression in these samples. GEMusicA includes a function for the unsupervised selection of differentially expressed genes on the basis of the variance. In the present paper we used this approach for the definition of a stem cell signature and tested the behavior of this signature in EFT microarray data.

## 2. Materials and Methods

### 2.1. Microarray Data Sets

All microarray data sets were downloaded from the Gene Expression Omnibus (GEO) database [15]. The following data sets were used: GSE1824 [1], GSE1825 [2], GSE7007 [3], GSE2248 [16], GSE2361 [17], GSM139881–GSM139883 and GSM139888–GSM139893 from GSE6029 [18], GSM86779, GSM86781 and GSM867783 form GSE3788 [19], GSE2638 [20], GSE2639 [20], and GSM1529859–GSM1529861 from GSE62600 [21].

### 2.2. GEMusicA Analysis

Cel files were processed with the GEMusicAR script [14]. In a first step, cel files from hematopoietic stem cells (HSC) [19], embryonic stem cells (ESC) [16], neuronal stem cells (NSC) [21], and different subtypes of mesenchymal stem cells (MSC) [3, 16, 18] were RMA (Robust Multiarray Average) normalized and used for selection of probe sets with differential expression between different stem cell types. The original GEMusicAR script [14] uses the RMA algorithm only in combination with Affymetrix Exon arrays. In order to allow usage with the Affymetrix HG_U133A microarrays in this paper, the argument “level” in the function “ProcessCelRMA” was deleted. The number of probe sets that were used for generation of melodies was set to 1114 (5% of the total number of probe sets on the used arrays). The 1114 probe sets that were filtered by GEMusicA in this way were used to generate a prefiltered probe set list that was used in the following steps for analysis of tumor samples. For these analyses, the cel files from stem cells were combined with the required tumor samples or normal tissue samples and RMA normalized. Subsequent transformations of signal intensities into frequencies were performed by using the complete number of prefiltered probe sets (*N* = 1114). Music scores were prepared from the automatically generated TeX documents as described [14].

## 3. Results and Discussion

**Figure 1:**
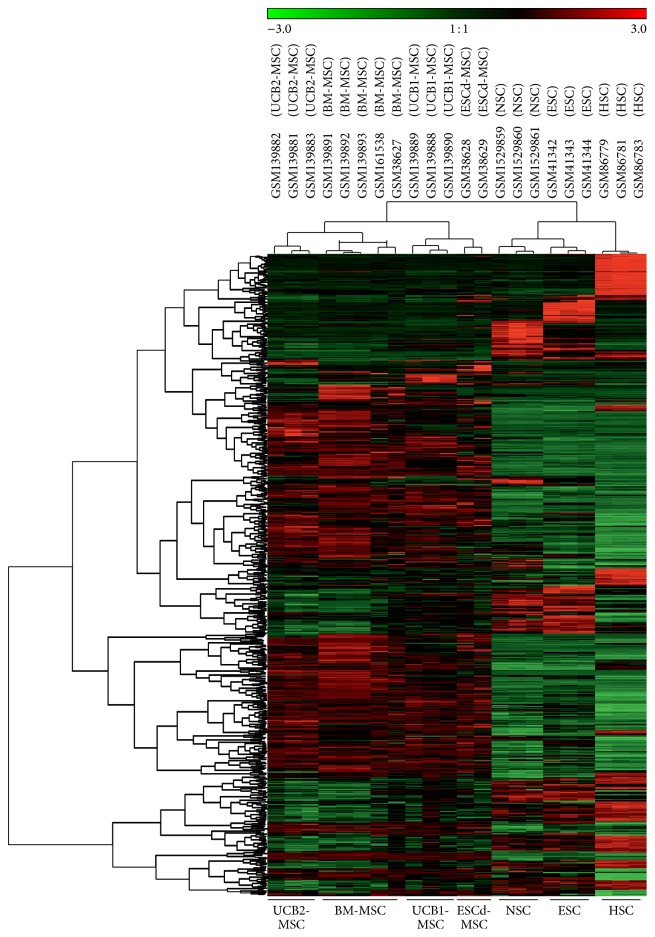
GEMusicA can discriminate between different stem cell types. DNA microarray cel files from embryonic stem cells (ESC) [16], neuronal stem cells (NSC) [21], hematopoietic stem cells (HSC) [19], bone marrow derived mesenchymal stem cells (BM-MSC) [3, 16, 18], two types of umbilical cord blood derived mesenchymal stem cells (UCB1-MSC, UCB2-MSC) [18], and mesenchymal stem cells derived from embryonic stem cells (ESCd-MSC) [16] were RMA-normalized and used for transformation into melodies using the GEMusicA implementation in R. The following parameters were used: minfreq = 27.5, tonesteps = 12, numkeys = 88, mindur = 8, vol = 4, TeXscalefactor = 1, maxNdots = 2, and *N* = 1114. Presented is a cluster analysis of the GEMusicA-processed 1114 probe sets (Manhattan distance, complete linkage clustering).

**Figure 2:**
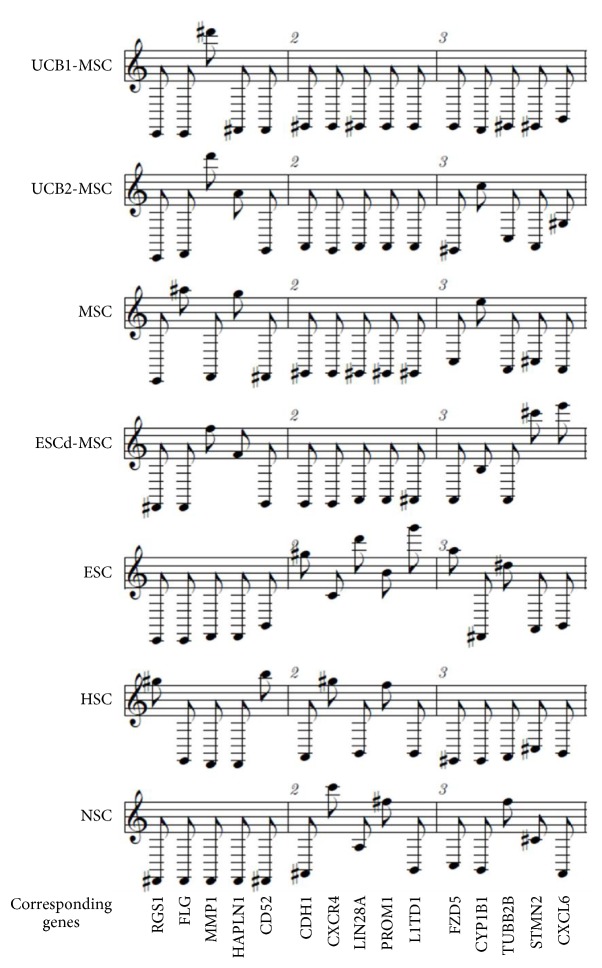
Stem cell melodies. DNA microarray cel files were processed as described in the caption of Figure 1. Presented are the first three bars from individual representative samples.

GEMusicA generates melodies from DNA microarray data. The algorithm includes a procedure that filters probe sets with high variance of the signal intensities. These probe sets are likely to have a higher information content than probe sets with low variability [14]. We used this approach for the characterization of tumor specific gene expression profiles and demonstrated that the generated melodies can be used for discrimination between different tumor entities, for example, neuroblastoma and Ewing sarcoma cell lines [14]. We asked whether this method can be used for the definition of gene expression signatures that are specific for certain stem cell populations. For this end we combined microarray data from embryonic stem cells, neuronal stem cells, hematopoietic stem cells, and different types of mesenchymal stem cells and used GEMusicA for the generation of melodies. As shown in Figure 1, the transformed signal intensities from the GEMusicA-filtered probe sets allow clear discrimination between the different stem cell types.  Figure 2 shows the first 15 tones from the melodies from representative samples. The corresponding genes include several well-known stem cell specific candidates. For example, embryonic stem cells are characterized by a high frequency of the tones representing LINE-1 type transposase domain containing 1 (L1TD1) or Lin-28 homolog A (LIN28A) that are both known to be markers for ESC [22, 23]. Similarly, prominin 1 (PROM1 = CD133) which is a marker for HSC [24] and NSC [25] is presented by high-pitched tones in the melodies from these two stem cell types (Figure 2).

**Figure 3:**
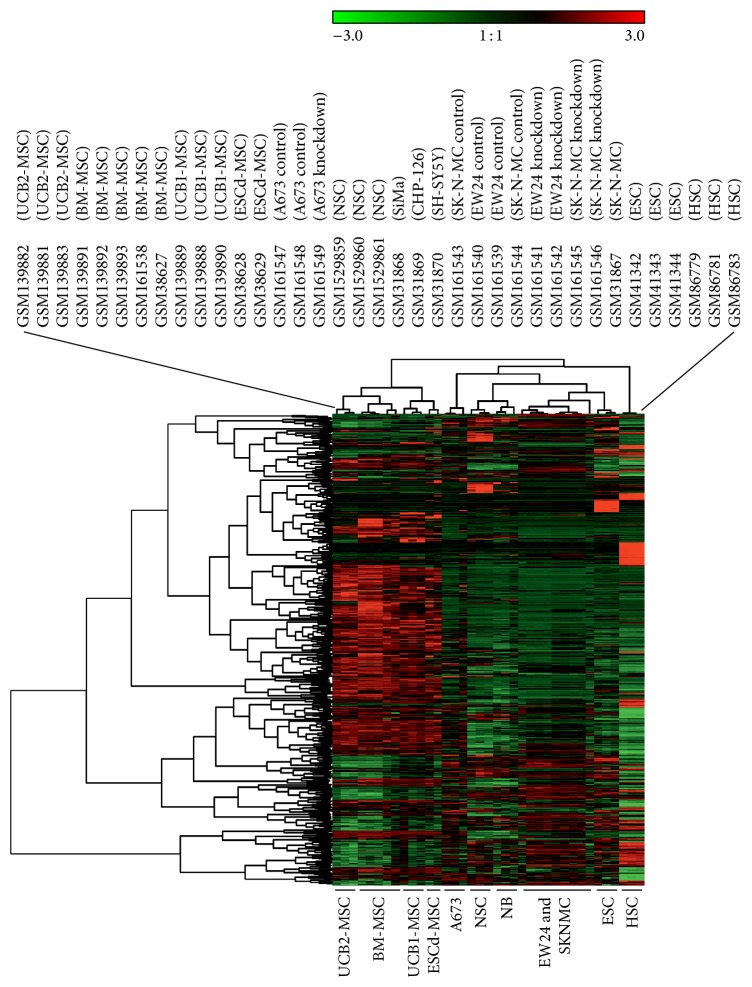
The stem cell signature of Ewing sarcoma cells. DNA microarray cel files from stem cells were processed as described in the caption of Figure 1. DNA microarray data from these stem cells were combined with data from a panel of neuroblastoma cell lines (NB) [1] and the Ewing sarcoma cell lines A673, EW24, and SK-N-MC [1, 3]. After RMA normalization, transformation into melodies was performed using the GEMusicA implementation in R. For this end, only the probe sets from the prefiltered stem cell signature from Figure 1 were used. The following parameters were used: minfreq = 27.5, tonesteps = 12, numkeys = 88, mindur = 8, vol = 4, TeXscalefactor = 1, maxNdots = 2, and *N* = 1114. Presented is a cluster analysis of the GEMusicA-processed 1114 probe sets (Manhattan distance, complete linkage clustering).

**Figure 4 fig4:**
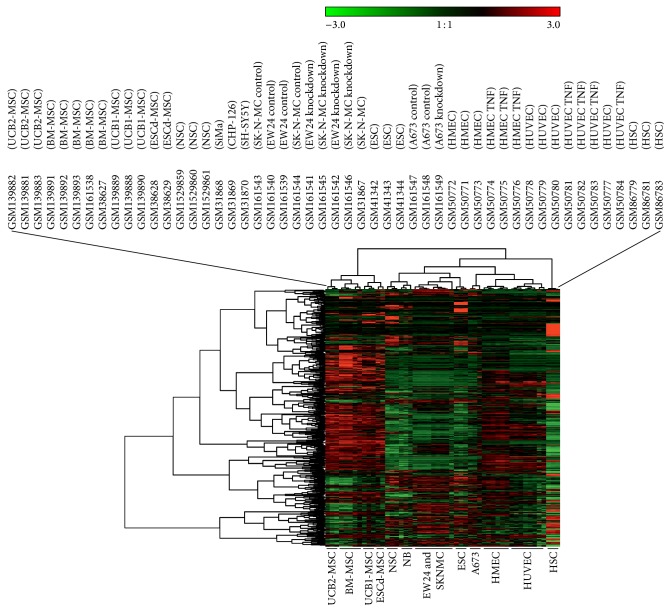
Similarities in the stem cell signature between Ewing sarcoma cells and endothelial cells. DNA microarray cel files from stem cells were processed as described in the caption of Figure 1. DNA microarray data from these stem cells were combined with a panel of microvasculature endothelial cells (HMEC) [20], macrovasculature endothelial cells (HUVEC) [20], neuroblastoma cell lines (NB) [1], and the Ewing sarcoma cell lines A673, EW24, and SK-N-MC [1, 3]. After RMA normalization, transformation into melodies was performed using the GEMusicA implementation in R. For this end, only the probe sets from the prefiltered stem cell signature from Figure 1 were used. The following parameters were used: minfreq = 27.5, tonesteps = 12, numkeys = 88, mindur = 8, vol = 4, TeXscalefactor = 1, maxNdots = 2, and *N* = 1114. Presented is a cluster analysis of the GEMusicA-processed probe sets (Manhattan distance, complete linkage clustering).

**Figure 5:**
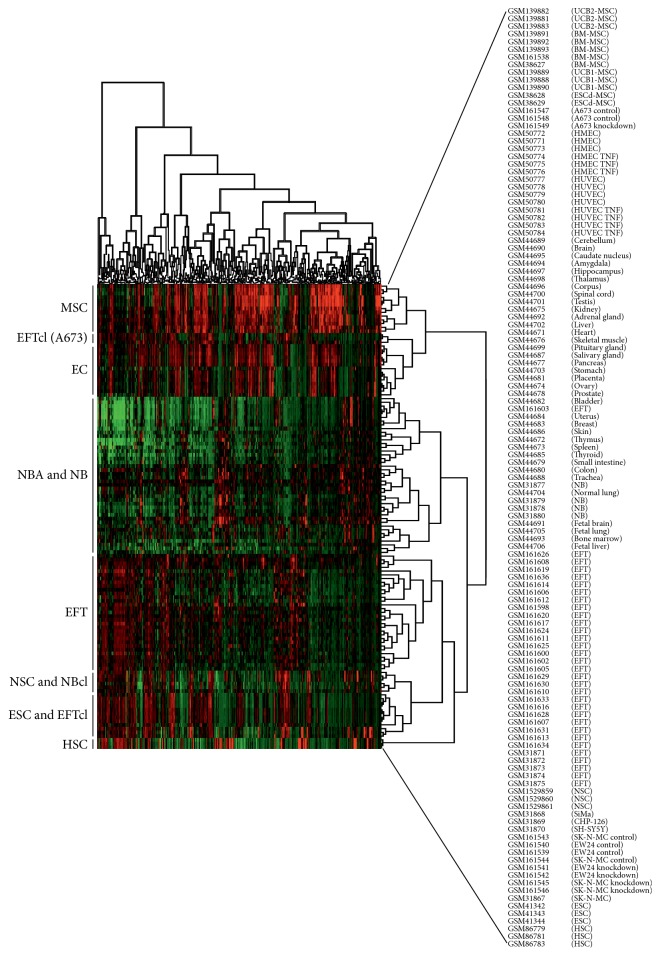
The stem cell signature of Ewing sarcoma biopsies. DNA microarray cel files from stem cells were processed as described in the caption of Figure 1. DNA microarray data from these stem cells were combined with a panel of microvasculature endothelial cells (HMEC) [20], microvasculature endothelial cells (HUVEC) [20], neuroblastoma cell lines (NBcl) [1], Ewing sarcoma cell lines A673, EW24, and SK-N-MC (EFTcl) [1, 3], neuroblastoma biopsies (NB) [2], Ewing sarcoma biopsies (EFT) [2, 3], and a panel of normal tissues of varying origin (NBA) [17]. Transformation into melodies was performed using the GEMusicA implementation in R. For this end, only the probe sets from the prefiltered stem cell signature from Figure 1 were used. The following parameters were used: minfreq = 27.5, tonesteps = 12, numkeys = 88, mindur = 8, vol = 4, TeXscalefactor = 1, maxNdots = 2, and *N* = 1114. Presented is a cluster analysis of the GEMusicA-processed probe sets (Manhattan distance, complete linkage clustering).

In a next step we included cell lines from Ewing sarcoma and neuroblastoma [1, 3] in the analysis (Figure 3). All EFT cell lines are characterized by EWS-FLI1 type 1 fusion transcripts. The neuroblastoma cell lines CHP-126 and SiMa are cell lines with a MYCN (v-myc avian myelocytomatosis viral oncogene, neuroblastoma derived) amplification whereas SH-SY5Y cells have no MYCN amplification. Neuroblastoma (NB) cell lines showed the expected high similarity to NSC. Interestingly, all EFT cell lines showed a higher similarity to NSC and ESC than to MSC (Figure 3). Cell lines SK-N-MC (established from a supraorbital metastasis of an Askin tumor) and EW24 (established from a bone tumor) formed even a cluster together with ESC, suggesting that the embryonic phenotype in these cell lines is more pronounced than in A673 cells (established from a muscle tumor). A relationship of EFT and endothelial cells has been considered for a long time [1, 4, 6]. Therefore, we asked how endothelial cells will behave in this analysis and we included a set of endothelial cells in our data set. As shown in Figure 4, the rough topology of the clustering tree remains unchanged. Interestingly, EFT cell line A673 formed a cluster together with the endothelial cells in this analysis whereas the other EFT cell line samples remained in the cluster with ESC. A673 cells have been shown to have endothelial differentiation capacity under certain conditions. After inhibition of enhancer of zeste homologue 2 or other components of the polycomb repressive complex 2 (PRC2), A673 cells start tube formation in matrigel assays [4]. The results from our GEMusicA analysis support the endothelial features of this cell line. After extension of the data set with native tumor biopsies from NB and EFT patients [1, 3] as well as a panel of normal tissues (normal body atlas, NBA [17]), we observed again the high similarity between A673 cells and endothelial cells (Figure 5). EFT biopsies clustered together with SK-N-MC cells, EW24 cells, NB cell lines, NSC, and ESC. NB biopsies showed a different behavior in this cluster analysis, indicating that the behavior of the EFT samples is specific for this tumor entity and not a general phenomenon for (small round blue cell) tumors. Four of five NB biopsies clustered together with fetal brain. The NB cell lines remained stable in the cluster with NSC.

**Figure 6:**
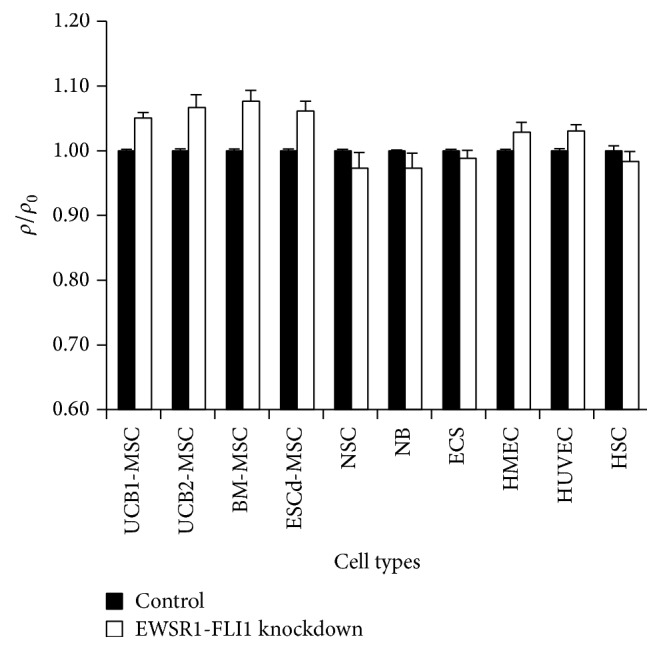
Relative Pearson correlation coefficients from the data from Figure 5. DNA microarray cel files were processed as described in the caption of Figure 5. Pearson correlation coefficients *ρ* were calculated for samples from Ewing sarcoma cell lines from data set GSE7007 [3] and all stem cell samples. For each EFT sample, the mean of the Pearson correlation coefficients in the control samples without EWSR1-FLI1 inhibition *ρ*_0_ was set as 1. Presented are means and standard deviations for the correlations between the indicated cell types and Ewing sarcoma cell lines after knockdown of EWSR1-FLI1 (EWS-FLI1 knockdown) or control cell lines (control) without EWSR1-FLI1 knockdown.

**Figure 7:**
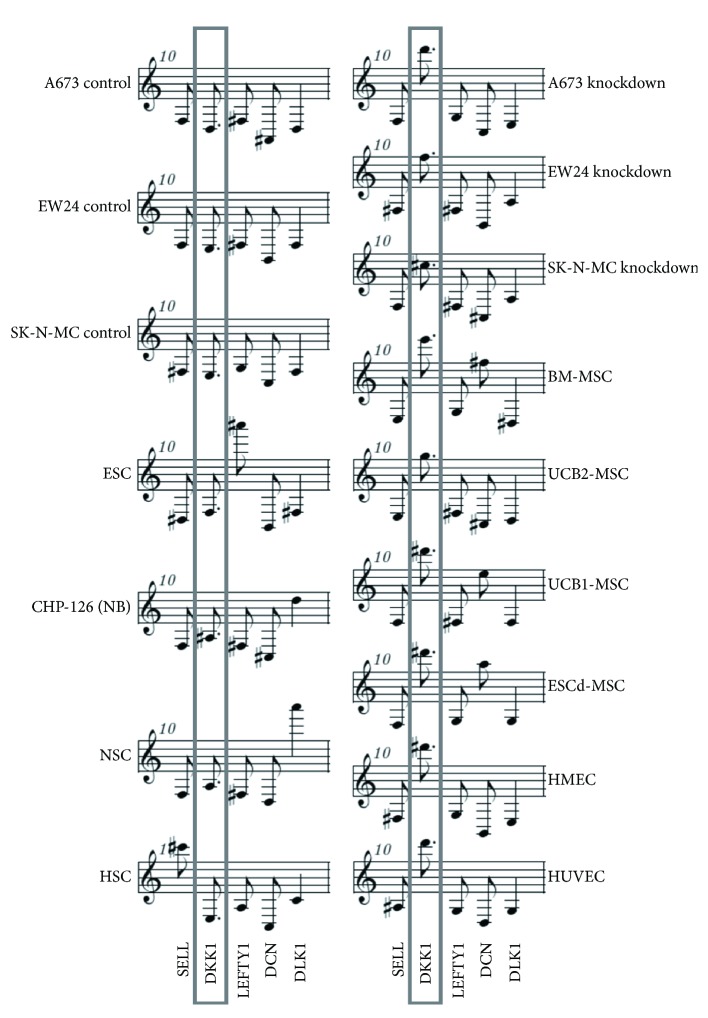
DKK1 expression discriminates between MSC/EC and other cell types. DNA microarray cel files were processed as described in the caption of Figure 5. Presented is the 10th bar from representative individual samples. The differentially expressed gene DKK1 is highlighted.

The high similarity between EFT cell lines and endothelial cells (A673) or ESC (EW24, SK-NM-C) can be a hint for histogenetic origin or a consequence of oncogene activation. The EFT cell line data set from GSE7007 contains data from EFT cell lines after knockdown of EWSR1-FLI1 [3]. In the cluster analyses (Figures 3 [Fig fig4]–5), EFT cell lines after knockdown of EWSR1-FLI1 and control cells clustered together, suggesting that EWSR1-FLI1 has only small impact on the expression of the genes in the defined stem cell signature. We compared the correlation coefficients between different non-EFT samples and EFT cell lines with and without knockdown of EWSR1-FLI1 (Figure 6). Interestingly, the correlation coefficients for ESC, NSC, HSC, or NB decreased after knockdown of EWSR1-FLI1 (*p* < 0.01) whereas the correlation coefficients for MSC or endothelial cells increased after knockdown of EWSR1-FLI1 (*p* < 0.00001). This increment in the correlation coefficient is based on changes in signal intensities for probe sets that can discriminate between MSC/endothelial cells and other stem cells. One example is shown in Figure 7. Dickkopf homolog 1 (DKK1) is upregulated in EWSR1-FLI1 inhibited EFT cells. DKK1 is highly expressed in MSC but also in endothelial cells as indicated by the high-pitched tones representing DKK1 (Figure 7). Downregulation of DKK1 and upregulation of DKK2 after transgenic expression of EWSR1-FLI1 in MSC have been described [26]. DKK2 which is an Ewing sarcoma specific gene [1] and is upregulated by EWSR1-FLI1 is not included in the prefiltered stem cell signature probe sets and, therefore, not included in the melodies. The decreased correlation coefficient between EFT and ESC after knockdown of EWSR1-FLI1 suggests that the expression of TET-ETS oncogenes in MSC can induce the expression of an ESC-like phenotype.

An interesting aspect of the presented GEMusicA analyses is the observation that different EFT cell lines behave differently. Whereas EW24 cells and SK-N-MC cells demonstrate a higher similarity to ESC, A673 cells show a very high similarity to endothelial cells (and MSC). In this regard it is interesting to note that A673 cells have been initially established as rhabdomyosarcoma cells [27] whereas SK-N-MC cells were established as neuroblastoma cell line [28], suggesting that the histological phenotype of EFT can vary extensively. Evidence for the classification of both cell lines as EFT comes from the detection of EWSR1-FLI1 fusions. Taking into account the fact that EWSR1-FLI1 can induce EFT-like gene expression profiles in different other cell types, it seems possible that different cells of origin can give raise to EFT. On the other hand, TET-ETS translocations are the sole cytogenetic aberration in only approximately one-fourth of EFT (data from the Mitelman Database of Chromosome Aberrations and Gene Fusions in Cancer, http://cgap.nci.nih.gov/Chromosomes/Mitelman). The large majority of tumors show additional alterations and it seems likely that at the molecular level 100% of the tumors will harbor “secondary” alterations. Therefore, it seems evident that TET-ETS fusions alone are not sufficient for the development of EFT and that additional events are required. These events might vary between different tumors and might be one reason for the heterogeneity of EFT cell lines. The histogenetic origin of EFT is a miracle for nearly a century. At diagnosis, the tumor cells have a history of* in vivo* growth and evolution that had molded the phenotype and gene expression of the tumor cells. These effects are at least partially independent of the TET-ETS oncogene. Therefore, ectopic expression of TET-ETS oncogenes in normal cells will not result in the exact EFT phenotype. On the other hand, manipulation of TET-ETS expression in EFT will likely not result in a cell with the exact phenotype of the cell of origin. From our data we cannot draw the conclusion that MSC are optimal candidates for EFT mother cells. Endothelial cells (or probably an endothelial precursor cell population) should be considered as an alternative source at least for a subpopulation of EFT.

## 4. Conclusions

Our data demonstrate that GEMusicA is feasible for the characterization of stem cell-type specific gene expression signatures. The comparison of GEMusicA-processed DNA microarray melodies from EFT and stem cells supports the concept of an endothelial and embryonic phenotype of the EFT mother cell.
